# Right‐sided diverticulitis

**DOI:** 10.1002/ccr3.2290

**Published:** 2019-07-01

**Authors:** Vincent Zimmer

**Affiliations:** ^1^ Department of Medicine Marienhausklinik St. Josef Kohlhof Neunkirchen Germany; ^2^ Department of Medicine II, Saarland University Medical Center Saarland University Homburg Homburg Germany

**Keywords:** abdominal pain, colonic diverticulosis, diverticulitis

## Abstract

Right‐sided diverticulitis is uncommon in Western populations with few to zero reports on endoscopic correlations. Although ultrasound or imaging studies typically detect advanced forms to be differentiated from acute appendicitis and/or Crohn’s disease, in more subtle presentations with an indication of urgent ileocolonoscopy, endoscopy may reliably establish an unequivocal diagnosis.

A 79‐year‐old woman presented for diffuse abdominal pain and hematochezia without hemodynamic compromise. Laboratory investigations indicated normal blood counts and raised C‐reactive protein levels (11.5 mg/L). Clinical examination revealed diffuse tenderness without peritoneal signs. Abdominal ultrasound demonstrated minor pandiverticulosis without definitive wall thickening. The patient underwent ileocolonoscopy the next day revealing an eclectic picture. In the ascending colon, focal inflammatory changes around a diverticulum near the ileocecal valve emerged suspicious for (multi‐) focal diverticulosis (Figure 1A). This was further substantiated by semilunar ulcerations around several other right colonic diverticula with high‐grade edematous changes (Figure 1B). Of interest, in the sigmoid, the diverticula openings were completely free of inflammatory signs while the interdiverticular mucosa exhibited erythematous and edematous findings with loss of vascular pattern consistent with segmental colitis associated with diverticulosis (Figure 2A). Concerning GI bleeding, flexible proctoscopy indicated necrotic mucosal defects on larger hemorrhoids indicating subacute hemorrhoidal bleeding (Figure 2B).

**Figure 1:**
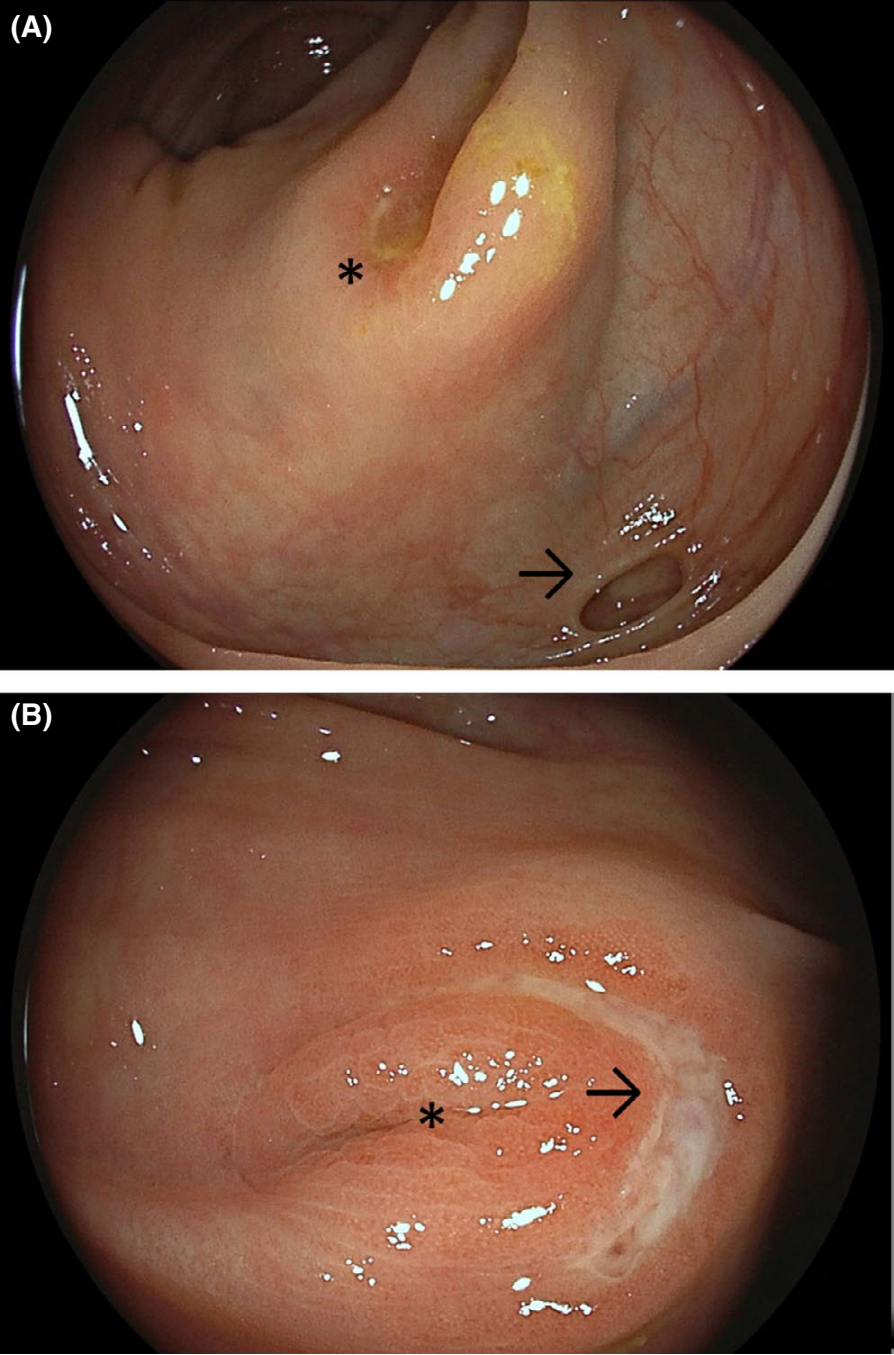
A, Endoscopic view of the ascending colon with a focally inflamed diverticulum close to the ileocecal valve (asterisk). Please also note the unremarkable adjacent diverticulum (arrow). B, illustrates a semilunar ulceration (arrow) around another right colonic diverticula with high‐grade edematous changes with the ensuing slit‐like collapse of the orificium (asterisk)

**Figure 2:**
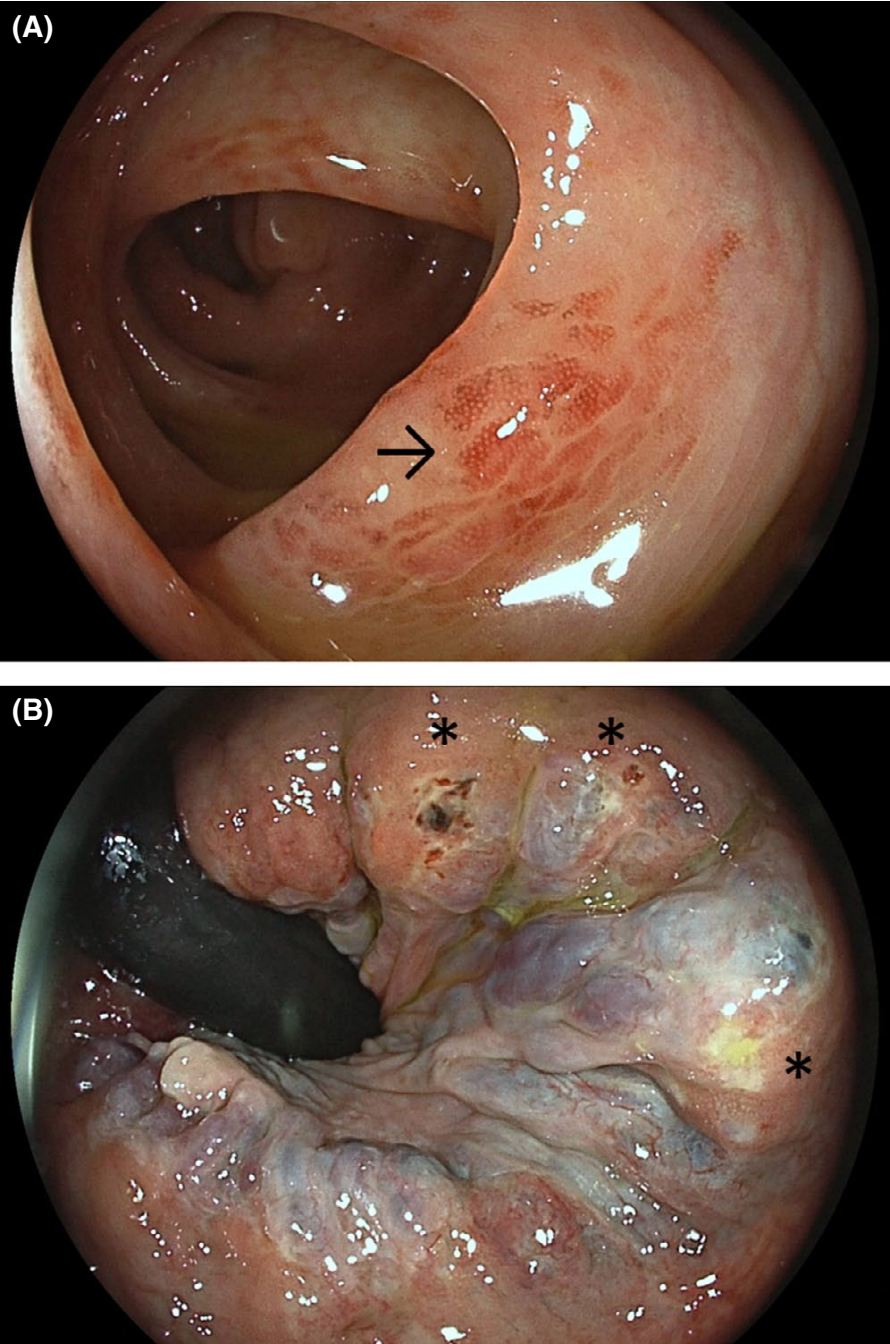
A, Inflammatory changes with erythema and edema (arrow) in the interdiverticular mucosa of the sigmoid with loss of vascular pattern consistent with segmental colitis associated with diverticulosis. B, Retroflexion in the rectum for flexible proctoscopy indicated partly necrotic mucosal defects (asterisks) indicating subacute hemorrhoidal bleeding

Right‐sided diverticulitis uncommon in Western populations, and there is little evidence to guide clinical decisions. Clinical differentiation from acute appendicitis and/or Crohn’s disease is important.^1^ However, treatment considerations should lean on sigmoid diverticulitis with symptomatic and/or, as in this case, antibiotic treatment with rapid clinical and laboratory recovery.

## CONFLICT OF INTEREST

Nothing to declare.

## AUTHOR CONTRIBUTION

VZ: involved in clinical care, drafting, and finalization of the manuscript.
